# Experimental investigation and multi-response design of FFF-printed PLA honeycomb sandwich panels for flexural performance and mass efficiency

**DOI:** 10.1371/journal.pone.0358969

**Published:** 2026-09-21

**Authors:** Balram Yelamasetti, Abhishek Agarwal, Mahender Thotakuri, I. Sri Phani Sushma, Jamyang Choden, Naveen Kumar P, Harikishor Kumar

**Affiliations:** 1 Department of Mechanical Engineering, Sreyas Institute of Engineering and Technology, Hyderabad, Telangana, India; 2 Department of Energy Technology, Tallinn University of Technology, Tallinn, Estonia; 3 Department of Mechanical and Aerospace Engineering, Indian Institute of Technology Hyderabad, Kandi, Telangana, India; 4 Department of Mechanical Engineering, JNTUK University College of Engineering Narasaraopet, Andhra Pradesh, India; 5 Department of Mechanical Engineering, College of Science and Technology, Royal University of Bhutan, Phuentsholing, Bhutan; 6 Trident Welding Research and Academy, Hyderabad, India; 7 Department of Mechanical engineering, MLR Institute of Technology, Hyderabad, India; University of Ljubljana Biotechnical faculty: Univerza v Ljubljani Biotehniska fakulteta, SLOVENIA

## Abstract

Lightweight sandwich structures produced by fused filament fabrication (FFF) require geometric designs that provide adequate mechanical performance without excessive material usage. However, the geometric parameters governing flexural response and structural mass can favour different design configurations, making response-specific parameter selection unsuitable when both requirements must be considered simultaneously. This study experimentally investigates the effects of honeycomb cell size (HCS), honeycomb cell-wall thickness (HCT), top face-sheet thickness (TFS), and bottom face-sheet thickness (BFS) on the flexural strength and mass of FFF-printed polylactic acid (PLA) honeycomb sandwich panels. Eighteen geometric configurations were fabricated according to a mixed-level Taguchi L18 orthogonal array and evaluated by three-point bending and mass measurement. Analysis of means (ANOM) was used to identify response-specific factor levels, while analysis of variance (ANOVA) quantified the relative contributions of the geometric parameters. HCS and HCT accounted for 45.9% and 34.1% of the modelled variation in flexural strength, respectively, whereas HCT and HCS accounted for 50.9% and 34.0% of the modelled variation in panel mass. Because the parameter settings favoured by maximum flexural strength differed from those favoured by minimum mass, the ANOM factor levels were combined with normalized ANOVA contributions in a contribution-weighted multi-response selection procedure. The resulting configuration, HCS = 6 mm, HCT = 1.08 mm, TFS = 1.18 mm, and BFS = 1 mm, produced a flexural strength of 13.21 MPa and a mass of 34.367 g in the validation experiment. Compared with the highest-strength L18 configuration, the selected design retained 90.54% of the measured flexural strength while reducing panel mass by 50.29%. The results indicate that the core-related variables accounted for most of the modelled variation in the two measured responses. Within the investigated design space, the contribution-weighted procedure was used as a study-specific means of reconciling the response-specific factor settings and selecting a candidate strength–mass compromise for experimental evaluation.

## Introduction

Fused filament fabrication (FFF) is widely used for producing polymer components with complex geometries, comparatively low material waste, and flexible control over internal architecture [1–5]. Its layer-wise manufacturing principle is particularly suitable for lightweight structural design because material distribution can be controlled through geometric modelling and process parameters. Polylactic acid (PLA) is among the most commonly used thermoplastics in FFF because of its processability, availability, and favourable specific mechanical properties [6,7]. However, the mechanical response of FFF-printed PLA components depends on both manufacturing conditions and structural geometry, including material distribution and build-related parameters [6,7]. When the printing conditions are held constant, structural geometry provides a direct means of modifying load-bearing behaviour and material usage. This makes geometric design particularly important when mechanical performance must be improved without an excessive increase in structural mass.

Cellular architectures provide an effective means of controlling the mechanical performance and mass of additively manufactured components. Among these architectures, hexagonal honeycomb structures are widely investigated because their response can be tailored through cell size, wall thickness, and face-sheet geometry [8,9]. In a sandwich panel subjected to bending, the face sheets primarily resist normal stresses, whereas the cellular core transfers shear loads and maintains separation between the faces. Consequently, modifications to the core and face-sheet dimensions can alter both the load-bearing behaviour and material requirement of the complete structure. Understanding these coupled effects is essential for the design of lightweight FFF-printed sandwich panels.

Previous investigations have established that the mechanical behaviour of additively manufactured cellular structures is sensitive to core topology, geometric dimensions, material distribution, and loading conditions. Montazeri et al. [10] examined conventional and auxetic 3D-printed honeycombs under three-point bending and demonstrated the influence of structural configuration and internal reinforcement on flexural response. Gajdoš et al. [11] reported differences in the flexural behaviour of FFF components produced with honeycomb and sparse internal structures, while Bharath et al. [12] demonstrated the potential of additively manufactured cellular sandwich composites under bending loads. These studies confirm the importance of internal architecture but also show that the mechanical response of cellular structures cannot be considered independently of their geometric design. The utility of honeycomb architectures has also been demonstrated in protective applications, where incorporation of a honeycomb lattice reduced the simulated impact response of an electric-vehicle battery-pack configuration [13].

Studies focused specifically on polymeric honeycomb sandwich structures have further demonstrated the influence of core and face-sheet parameters. Brischetto and Torre [14] experimentally examined FFF-printed PLA honeycomb sandwich specimens and reported the dependence of structural response on material and core configuration. Hashemi and Galehdari [15] investigated functionally graded PLA honeycombs and showed that variations in cellular geometry considerably influence mechanical response and energy absorption. Cojocaru et al. [16] compared hexagonal, grid, and triangular PLA structures under bending, demonstrating the effects of topology and loading orientation. Antony et al. [17] examined hemp fibre/PLA honeycomb sandwich panels and demonstrated the potential of reinforced FFF structures for lightweight applications.

Experimental design and statistical analysis provide a practical means of separating the effects of multiple design variables without requiring exhaustive testing of every possible parameter combination. Taguchi methods have been applied to cellular and additively manufactured structures to evaluate geometric and manufacturing variables using reduced experimental programmes [18,19]. More recent investigations have addressed the structural optimization and mechanical performance of additively manufactured sandwich systems [20–22]. Recent work has also demonstrated the potential of biomimetic honeycomb architectures for balancing lightweight construction with structural and functional performance, further illustrating the versatility of honeycomb-based designs [23]. Geramizadeh et al. [24] specifically examined face-sheet thickness in polymeric honeycomb sandwich beams, demonstrating that face-sheet design can influence bending behaviour and should be considered together with core geometry.

Despite these developments, an important design issue remains when flexural performance and structural mass must be considered simultaneously in FFF-printed honeycomb sandwich structures. Previous studies have shown that manufacturing parameters influence the mechanical response of additively manufactured polymers [25–27], while investigations of additively manufactured honeycomb and sandwich structures have demonstrated the importance of core architecture, cellular geometry, and face-sheet configuration [28]. Optimization studies have further shown the potential of systematic parameter-selection approaches for improving the performance of additively manufactured structures [29, 30]. However, the combined effects of honeycomb cell size, cell-wall thickness, and the two face-sheet thicknesses on both flexural strength and total panel mass have received comparatively limited experimental attention within a single design framework. In particular, the factor levels that favour maximum flexural strength need not coincide with those that minimize mass, creating a need for a transparent procedure for selecting a practical compromise configuration.

The present study addresses this design requirement by experimentally evaluating four geometric variables-honeycomb cell size (HCS), honeycomb cell-wall thickness (HCT), top face-sheet thickness (TFS), and bottom face-sheet thickness (BFS)-within a mixed-level Taguchi L18 design. ANOM is used to identify the factor levels favoured independently by maximum flexural strength and minimum mass, while ANOVA is used to quantify the relative contribution of each parameter to the two responses. The response-specific factor settings are subsequently combined with the normalized ANOVA contributions to obtain a contribution-weighted compromise configuration, which is then fabricated and experimentally evaluated.

The study is therefore intended to answer three design questions: which geometric parameters predominantly control flexural strength and mass; whether the same parameter settings can satisfy the two competing responses; and whether contribution-weighted combination of the response-specific settings can produce a practically useful lightweight configuration. Accordingly, the objectives are to (i) quantify the effects of HCS, HCT, TFS, and BFS on measured flexural strength and panel mass; (ii) determine the statistical contribution and significance of each parameter; (iii) identify and reconcile the conflicting factor settings associated with the two responses; and (iv) experimentally evaluate the resulting compromise configuration.

The principal contribution of this work is not the use of Taguchi analysis or ANOVA individually, but the integration of experimentally obtained factor-level trends and response-specific contribution ratios in the selection of a single strength–mass compromise design. This provides a transparent link between experimental sensitivity analysis and subsequent parameter selection while avoiding the assumption that the configuration favoured by either response alone represents a suitable lightweight design.

## Materials and methods

### Material and fabrication conditions

Commercial polylactic acid (PLA) filament with a nominal diameter of 1.75 mm was used to fabricate all specimens. The PLA filament was manufactured by eSUN. The filament was received directly from the supplier and used as received for printing the honeycomb structures, without any additional drying or reheating treatment. The extrusion temperature, nominal infill density, layer thickness, printing speed, and bed temperature were 210 °C, 100%, 0.2 mm, 30 mm/s, and 55 °C, respectively. The cooling fan was switched off during printing. The wall/perimeter count and extrusion width were not recorded. All panels were printed in a flat orientation, with the bottom face placed directly on the build plate. The longitudinal direction of the panel was aligned with the X-axis of the build platform, while the honeycomb cell walls were built vertically along the Z-axis. This orientation was maintained for all configurations to ensure a common build direction and consistent layer arrangement during comparative evaluation; alternative build orientations were not investigated in the present study. These fabrication conditions were kept unchanged throughout the experimental programme so that the analysis focused on the selected geometric variables. The available records therefore do not permit reconstruction of the complete slicer toolpath settings, although the recorded printing conditions were kept unchanged for all experimental configurations.

### Specimen geometry and experimental design

The honeycomb sandwich panels were modelled using Autodesk Fusion 360 (2024) and exported in stereolithography (STL) format. The models were processed using Simplify3D (Version 5.1) to generate the G-code and were fabricated using a PRATHAM 5.0 FFF machine (MAKE3D.IN) equipped with a 0.4 mm nozzle.

Four geometric parameters were investigated: HCS, HCT, TFS, and BFS. HCS and HCT control the geometry and material distribution of the cellular core, whereas TFS and BFS control the material distribution in the face sheets. The 1, 2, and 3 mm TFS and BFS levels represent the nominal face-sheet thicknesses specified in the CAD models rather than prescribed numbers of perimeter lines. The available records do not contain the extrusion-width or perimeter-count settings used during slicing to realize these nominal dimensions. Likewise, the intermediate HCT and TFS dimensions used for the validation configuration are reported as nominal CAD dimensions; no claim is made that the corresponding as-built dimensions were identical to the nominal values. These variables were selected because changes in their dimensions can simultaneously affect load transfer, resistance to deformation, and the quantity of material required for fabrication [4,10,20–22]. A conventional hexagonal topology was retained for all specimens so that the experimental analysis focused on the selected dimensional variables rather than changes in cellular topology. The investigated parameter levels are given in Table 1.

**Table 1:** Geometric design parameters and factor levels used in the experimental design.

Parameter	Symbol	Level 1	Level 2	Level 3
Honeycomb cell size (mm)	HCS (A)	6	8	–
Honeycomb cell-wall thickness (mm)	HCT (B)	1	1.5	2
Top face-sheet thickness (mm)	TFS (C)	1	2	3
Bottom face-sheet thickness (mm)	BFS (D)	1	2	3

HCS was investigated at two levels, whereas HCT, TFS, and BFS were each investigated at three levels. A mixed-level L18 Taguchi orthogonal array was therefore selected to organize the experimental programme [18,19]. The design comprised 18 geometric configurations and enabled the effects of the four parameters on flexural strength and mass to be systematically evaluated with a reduced experimental set. One specimen was tested for each of the 18 L18 configurations, and one specimen was tested for the confirmation configuration. The values reported in Table 2 therefore represent individual experimental measurements rather than replicated mean values. The factor combinations and measured responses are presented in Table 2.

**Table 2:** Taguchi L18 experimental design, specimen dimensions, measured responses, and flexural-strength-to-mass ratio.

S. No.	HCS (mm)	HCT (mm)	TFS (mm)	BFS (mm)	TL (mm)	TH (mm)	TW (mm)	SL (mm)	SL/TH	Flexural strength (MPa)	Mass (g)	Apparent density (g/cm³)	Flexural strength/Mass (MPa/g)
1	6	1	1	3	157	36	20	109	3.0	4.78	39.29	0.348	0.122
2	6	1	2	2	157	36	20	109	3.0	5.58	39.29	0.348	0.142
3	6	1	3	1	157	36	20	109	3.0	7.00	39.29	0.348	0.178
4	6	1.5	1	3	157	37	20	110	3.0	6.19	50.57	0.435	0.122
5	6	1.5	2	2	157	37	20	110	3.0	6.97	50.57	0.435	0.138
6	6	1.5	3	1	157	37	20	110	3.0	10.38	50.57	0.435	0.205
7	6	2	1	2	157	36	20	109	3.0	12.78	57.53	0.509	0.222
8	6	2	2	1	157	36	20	109	3.0	11.17	57.53	0.509	0.194
9	6	2	3	3	157	39	20	118	3.0	14.59	69.13	0.565	0.211
10	8	1	1	1	209	45	20	134	3.0	2.52	42.41	0.225	0.059
11	8	1	2	3	209	48	20	143	3.0	3.70	57.80	0.288	0.064
12	8	1	3	2	209	48	20	143	3.0	3.54	57.80	0.288	0.061
13	8	1.5	1	2	209	46	20	138	3.0	2.84	62.95	0.327	0.045
14	8	1.5	2	1	209	46	20	138	3.0	3.00	62.95	0.327	0.048
15	8	1.5	3	3	209	49	20	147	3.0	5.16	78.36	0.383	0.066
16	8	2	1	1	209	46	20	137	3.0	4.62	72.75	0.378	0.064
17	8	2	2	3	209	49	20	146	3.0	5.34	88.19	0.431	0.061
18	8	2	3	2	209	49	20	146	3.0	6.95	88.19	0.431	0.079

Note- Envelope-based apparent density was calculated as the measured panel mass divided by the external specimen volume (TL × TW × TH). This quantity is used only as a geometric normalization metric and does not represent the intrinsic density of the PLA material. Flexural strength/mass was calculated as the measured flexural strength divided by the measured panel mass and is reported as a supplementary descriptive metric.

**Fig 1:**
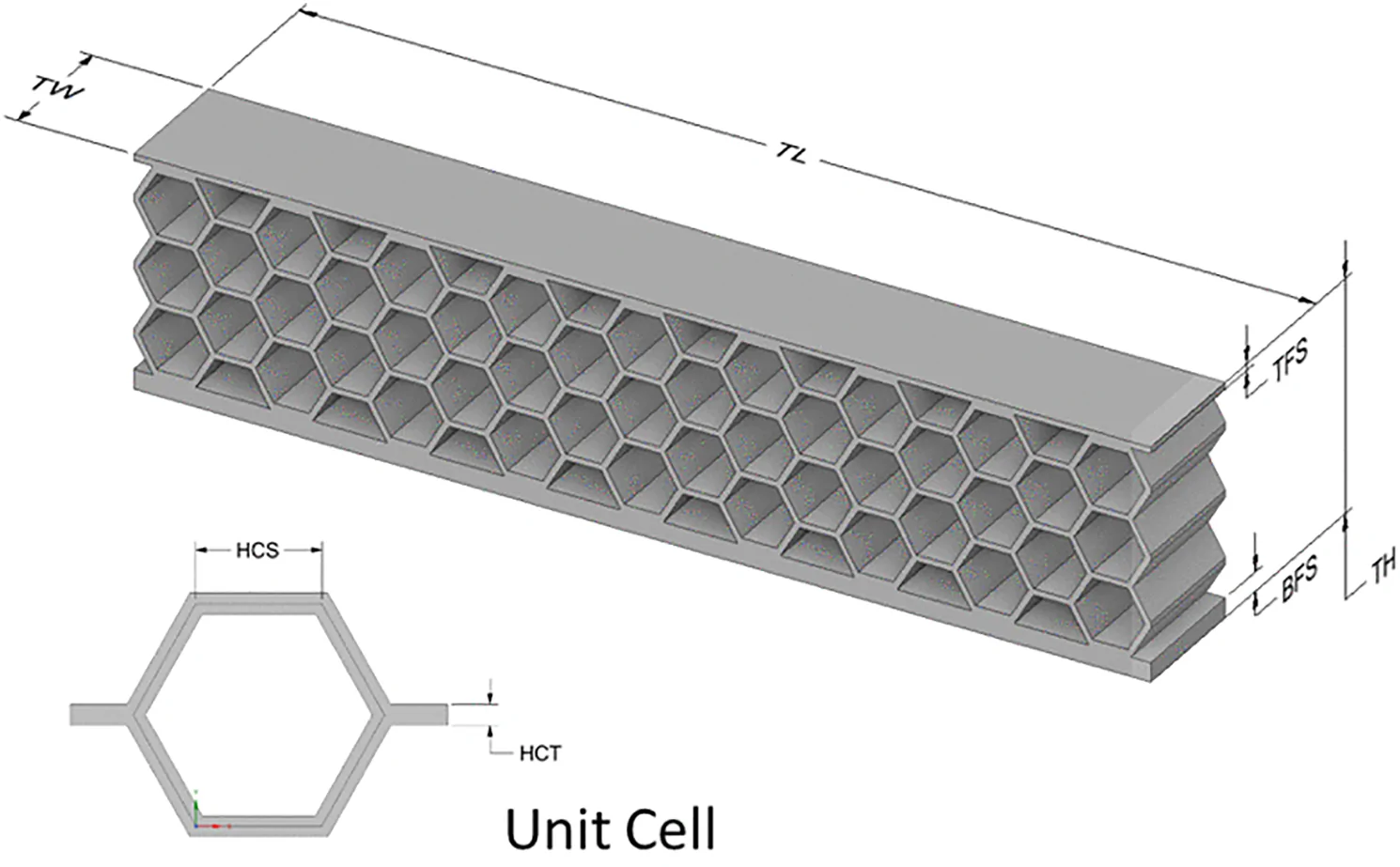
Geometry and principal dimensions of the honeycomb sandwich panel and hexagonal unit cell.

Each specimen consisted of a conventional hexagonal honeycomb core integrated with top and bottom face sheets. The overall specimen dimensions resulting from the investigated geometric configurations are reported in Table 2. The cellular count and topology were kept consistent while HCS was varied; consequently, changing HCS also changed the overall specimen length and, through the fixed nominal span-to-height condition, the support span. Therefore, the HCS contribution should be interpreted as the response of the complete geometric configuration rather than as an isolated cell-size effect. This design feature was considered when interpreting the ANOVA contribution of HCS. As illustrated in Fig 1, the overall specimen length, width, and height are denoted by TL, TW, and TH, respectively, while HCS and HCT represent the cell size and cell-wall thickness.

### Measurement of responses

**Fig 2:**
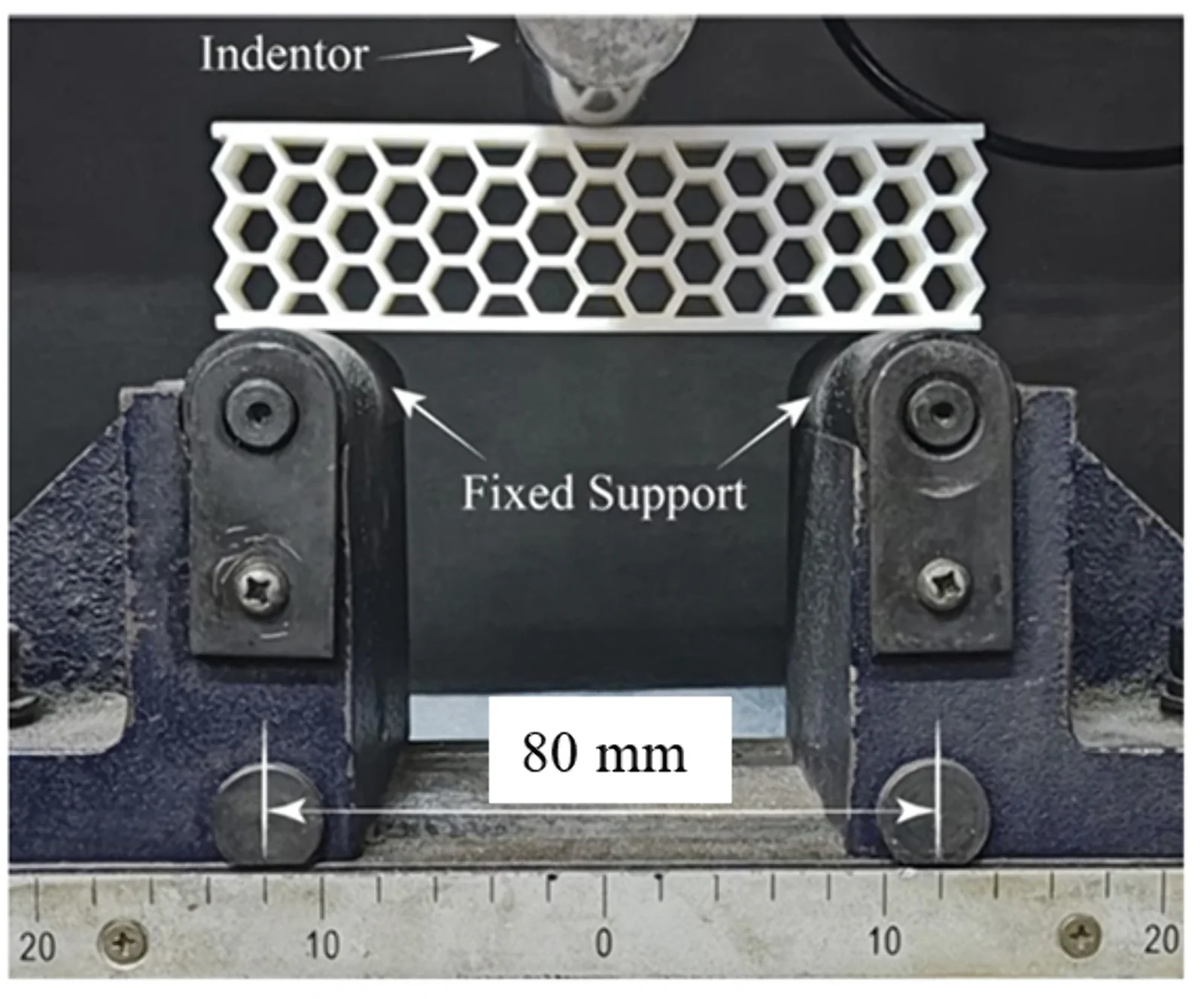
Experimental arrangement used for three-point bending of the honeycomb sandwich panels.

Three-point bending tests were conducted with reference to the test arrangement described in ASTM D7249/D7249M-20 using a universal testing machine equipped with a 10 kN load cell [31]. The approximately 3:1 span-to-height ratio used in the present study was a study-specific comparative condition and was not adopted as a material-level requirement of the standard. The same nominal ratio was maintained across the L18 configurations to provide a common comparative test condition despite differences in specimen height. The specimens were positioned on two supports and loaded centrally, as illustrated in Fig 2. Because the geometric configurations produced different specimen heights, the support span was adjusted to maintain a nominal span-to-height ratio of approximately 3, as reported in Table 2. Loading was applied at a constant crosshead displacement rate of 2 mm/min using a cylindrical loading nose with a diameter of 30 mm. The support span was selected to maintain a consistent nominal span-to-height ratio across the different specimen heights. The approximately 3:1 ratio was retained as a common comparative test condition across the different specimen heights rather than being used to establish a material-level flexural property. Because the resulting span-to-height ratio was approximately 3, the measured response may include contributions from core shear and local deformation near the loading and support regions in addition to face-sheet bending. The post-test photographs were used to document the visible deformation of the fabricated panels. Because the available experimental record does not contain a systematic failure-mode classification or close-up measurements of the damaged regions, individual specimens were not assigned to specific failure mechanisms. The mechanical response is therefore discussed in terms of the overall sandwich behaviour and the possible contributions of core deformation and local loading-region effects. Accordingly, the reported value is interpreted as the nominal flexural response of the complete sandwich configuration under the specified three-point bending arrangement rather than as a pure material property. The selected span-to-height ratio was applied consistently across the experimental configurations for comparative evaluation and was not intended to establish a material-level flexural property. The load–displacement response was recorded during each test, and the maximum load was used to obtain the reported nominal flexural-strength response. The original calculation sheet containing the individual maximum-load values and the exact flexural-strength calculation expression was not available among the experimental records accessible for this revision. Consequently, the individual maximum-load values and the original calculation expression cannot be reproduced from the surviving experimental documentation, and no additional calculation assumptions have been introduced in the present revision. The reported values are therefore retained as the experimentally obtained nominal flexural-strength responses. The mass of each fabricated panel was measured using an electronic balance with a stated resolution of 0.01 g. Flexural strength was considered as a larger-the-better response, whereas mass was treated as a smaller-the-better response for subsequent statistical analysis. These responses were selected to represent the competing requirements of mechanical performance and material efficiency.

## Results and discussion

**Fig 3:**
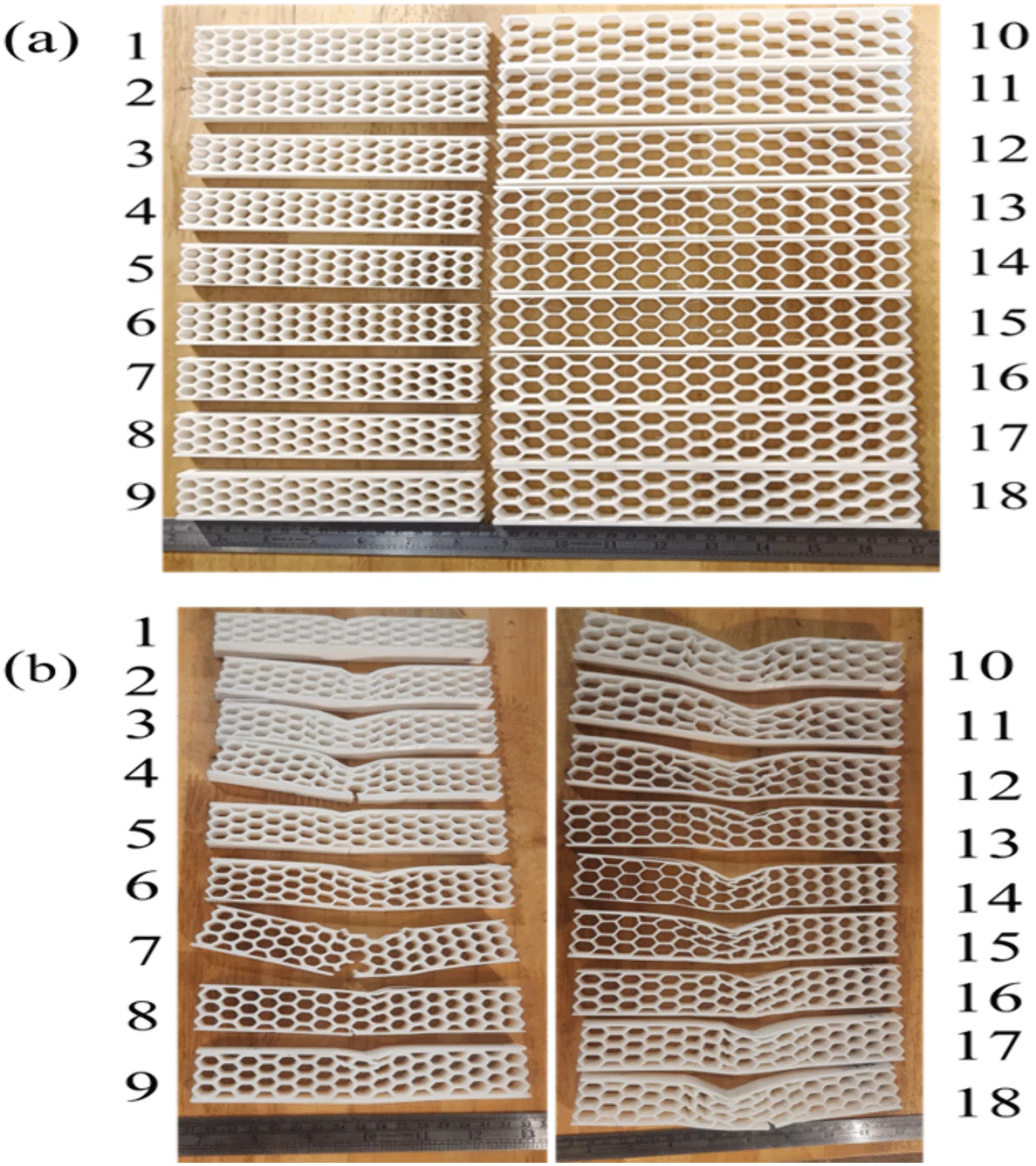
Honeycomb sandwich specimens. (a) before and (b) after three-point bending; numbers indicate the corresponding experiments listed in Table 2.

The flexural strength and mass measured for the 18 configurations are presented in Table 2. The tested specimens before and after three-point bending are shown in Fig 3. Considerable differences were observed across the experimental configurations, with flexural strength ranging from 2.52 to 14.59 MPa and mass ranging from 39.29 to 88.19 g.

Experiment 9 produced the highest measured flexural strength of 14.59 MPa with HCS = 6 mm, HCT = 2 mm, TFS = 3 mm, and BFS = 3 mm. In contrast, Experiment 10 produced the lowest measured flexural strength of 2.52 MPa with HCS = 8 mm, HCT = 1 mm, TFS = 1 mm, and BFS = 1 mm. These results indicate that smaller cells and thicker cell walls generally favoured flexural performance within the investigated design space. The subsequent ANOM and ANOVA results provide a quantitative assessment of these effects. The measured results demonstrate that geometric modifications influencing flexural performance also substantially affect structural mass, confirming the need for simultaneous consideration of both responses during parameter selection.

The lowest measured mass, 39.29 g, was obtained for Experiments 1–3, which shared HCS = 6 mm and HCT = 1 mm. Experiments 17 and 18 produced the highest mass of 88.19 g and shared HCS = 8 mm and HCT = 2 mm. The response values therefore demonstrate that geometric changes affecting flexural performance also substantially alter panel mass, confirming the need to consider the two responses simultaneously rather than selecting a configuration from either response alone. Because the honeycomb core and face sheets were fabricated as an integrated structure, the available measurements represent the mass of the complete panel; separate experimental masses of the core, top face sheet, and bottom face sheet were not recorded. The effects of TFS and BFS on total panel mass were therefore assessed through the ANOVA of the complete fabricated specimens.

### Analysis of means

Analysis of means (ANOM) was used to evaluate the average response at each factor level and identify the parameter settings associated with the preferred response for each objective [19,32]. Flexural strength was treated as a response to be maximized, whereas mass was treated as a response to be minimized. The ANOM results are presented in Tables 3 and 4. For flexural strength, the largest level effects were observed for HCT and HCS, with delta values of 4.722 and 4.641, respectively. TFS showed a smaller effect, while BFS produced only a minor variation in the mean response. Based on the highest mean flexural strength at each factor level, the response-specific configuration was HCS = 6 mm, HCT = 2 mm, TFS = 3 mm, and BFS = 3 mm. For minimum mass, the preferred levels were HCS = 6 mm, HCT = 1 mm, TFS = 1 mm, and BFS = 1 mm. HCT produced the largest variation in mean mass, followed by HCS, whereas TFS and BFS exhibited equal delta values. The different parameter settings associated with the two response objectives indicate a trade-off between flexural performance and panel mass and provide the basis for the subsequent multi-response analysis.

**Table 3:** ANOM response table for flexural strength.

Level	HCS: Honeycomb cell size	HCT: Honeycomb cell thickness	TFS: Top face sheet thickness	BFS: Bottom face sheet thickness
1	**8.825**	4.518	5.621	**6.626**
2	4.184	5.756	5.958	6.443
3	---	**9.240**	**7.935**	6.445
Delta	4.641	4.722	2.314	0.182
Rank	2	1	3	4

**Table 4:** ANOM response table for panel mass.

Level	HCS: Honeycomb cell size	HCT: Honeycomb cell thickness	TFS: Top face sheet thickness	BFS: Bottom face sheet thickness
1	**50.42**	**45.98**	**54.25**	63.89
2	67.93	59.33	59.39	59.39
3	---	72.22	63.89	**54.25**
Delta	17.51	26.24	9.64	9.64
Rank	2	1	3.5	3.5

### Analysis of variance

Analysis of variance (ANOVA) was performed to quantify the relative contribution and statistical significance of each geometric parameter [19,32]. The present analysis was restricted to the main effects of HCS, HCT, TFS, and BFS; factor interactions were not independently estimated in the selected L18 arrangement. Accordingly, the reported contributions should be interpreted as main-effect contributions within the fitted model rather than as fully independent physical effects. Because only one specimen was tested for each L18 configuration, the residual term should not be interpreted as an independent estimate of experimental repeatability; it may also contain unmodelled interactions and other sources of variation. The reported p-values therefore describe the fitted main-effects model for the available experimental observations. The ANOVA results for flexural strength are presented in Table 5. HCS and HCT were statistically significant at the 5% significance level, with p-values below 0.001 and equal to 0.001, respectively. TFS showed a smaller contribution and a borderline p-value of 0.053, whereas BFS was statistically insignificant within the investigated design space (p = 0.972). Because only one specimen was tested for each L18 configuration, standard deviations and coefficients of variation could not be calculated, and the residual term was not interpreted as a measure of experimental repeatability.

**Table 5:** ANOVA results for flexural strength.

Source	DoF	Seq. SS	Contribution	Adj. SS	Adj. MS	F-Value	P-Value
HCS	1	96.927	45.92%	96.9266	96.9266	41.50	<0.001
HCT	2	71.929	34.07%	71.9291	35.9646	15.40	0.001
TFS	2	18.750	8.88%	18.7498	9.3749	4.01	0.053
BFS	2	0.132	0.06%	0.1318	0.0659	0.03	0.972
Error	10	23.356	11.06%	23.3557	2.3356	–	–
**Total**	17	211.093	100.00%	–	–	–	–

#### Influence of geometric parameters on flexural strength.

Within the present experimental design, HCS was associated with the largest contribution to flexural strength, accounting for 45.92% of the modelled variation. The ANOM results show that reducing HCS from 8 to 6 mm increased the ANOM level-average response from 4.184 to 8.825 MPa. Smaller cells can provide more load-transfer paths within the core and may improve resistance to local deformation and core shear under bending. The observed trend is consistent with previous studies reporting a strong dependence of flexural behaviour on honeycomb geometry [10,11]. However, because changing HCS also changed the overall specimen length and support span in the present design, its contribution represents the response of the corresponding complete specimen geometry rather than an isolated cell-size effect. Accordingly, the 45.92% contribution should not be interpreted as the isolated physical contribution of cell size alone.

HCT was the second dominant parameter, contributing 34.07% of the modelled variation in flexural strength. Increasing HCT from 1 to 2 mm increased the ANOM level-average response from 4.518 to 9.240 MPa. The increase is consistent with the greater amount of load-bearing material in the core and the resulting resistance to cell-wall deformation under bending. The combined contributions of HCS and HCT reached 79.99%; however, this value should be interpreted within the present experimental design because the HCS levels were accompanied by changes in overall specimen geometry. TFS contributed 8.88% to the modelled variation in flexural strength and showed a progressive increase in mean response from 5.621 MPa at 1 mm to 7.935 MPa at 3 mm. However, its p-value of 0.053 does not meet the conventional 5% significance threshold, and the observed effect should therefore be interpreted cautiously. BFS contributed only 0.06% and was statistically insignificant (p = 0.972). The limited BFS effect is specific to the investigated geometry, loading arrangement, and parameter range and should not be interpreted as evidence that the bottom face sheet is generally unimportant in sandwich structures. The difference between the TFS and BFS effects on flexural strength is therefore specific to their positions within the tested sandwich configuration and loading arrangement, whereas their similar contribution to mass is expected because increasing either face-sheet thickness directly increases the amount of printed material. The comparatively smaller face-sheet contributions should also be interpreted in the context of the present sandwich configuration and the approximately 3:1 span-to-height ratio, for which the measured response may include contributions from core shear and local deformation in addition to face-sheet bending. Overall, the results indicate that the core-related variables accounted for substantially more of the modelled variation in measured flexural strength than the face-sheet thickness variables within the investigated design space.

#### Influence of geometric parameters on panel mass.

The ANOVA results for panel mass are presented in Table 6. HCT was the dominant parameter, accounting for 50.85% of the modelled variation, followed by HCS with a contribution of 33.98%. TFS and BFS contributed 6.87% and 6.87%, respectively, while the residual term accounted for 1.42%. Within the fitted main-effects model, all four geometric parameters showed statistically significant effects on panel mass at the 5% significance level. The strong influence of HCT is consistent with the direct increase in material volume produced by thicker cell walls throughout the honeycomb core. The ANOM results show that the mean mass increased from 45.98 g at HCT = 1 mm to 72.22 g at HCT = 2 mm. HCS also substantially affected mass, with the mean response increasing from 50.42 g at 6 mm to 67.93 g at 8 mm. This result should be interpreted together with the changing overall specimen dimensions reported in Table 2 because the 8 mm cell configurations had greater overall lengths than the 6 mm configurations. TFS and BFS produced equal ANOVA contributions to mass. Within the experimental design, both parameters altered the quantity of material contained in the outer faces and therefore affected panel mass. However, their contributions were considerably smaller than those of HCT and HCS. Overall, the ANOVA results presented in Table 6 show that the core-related variables dominated the variation in panel mass, reinforcing the need to account for the competing effects of geometry on both mechanical performance and material usage. Because the overall panel height varied among the investigated configurations, a given face-sheet thickness did not represent an identical fraction of the total panel thickness in every specimen. The TFS and BFS effects therefore correspond to the absolute nominal thickness levels specified in the experimental design rather than to constant face-sheet-to-panel-thickness ratios.

**Table 6:** ANOVA results for panel mass.

Source	DoF	Seq. SS	Contribution	Adj. SS	Adj. MS	F-Value	P-Value
HCS	1	1380.52	33.98%	1380.52	1380.52	239.15	<0.001
HCT	2	2065.56	50.85%	2065.56	1032.78	178.91	<0.001
TFS	2	279.20	6.87%	279.20	139.60	24.18	<0.001
BFS	2	279.20	6.87%	279.20	139.60	24.18	<0.001
Error	10	57.73	1.42%	57.73	5.773	–	–
**Total**	**17**	**4062.22**	**100.00%**	–	–	–	–

The ANOVA results for panel mass are presented in Table 6. HCT was the dominant parameter, accounting for 50.85% of the modelled variation, followed by HCS with a contribution of 33.98%. TFS and BFS contributed 6.87% each, while the residual term accounted for 1.42%. Within the fitted main-effects model, all four geometric parameters showed statistically significant effects on panel mass at the 5% significance level. The combined contribution of HCT and HCS to panel mass was 84.83%, compared with a combined contribution of 13.74% from TFS and BFS. Because the HCS levels also produced different overall specimen lengths, total panel mass cannot be interpreted as a geometry-independent measure of material efficiency. The present analysis therefore treats mass as the measured material-use response of the fabricated configurations. To provide an additional geometric normalization, envelope-based apparent density was calculated from the measured panel mass and external specimen volume (TL × TW × TH). This metric accounts for differences in overall specimen dimensions and is used only for comparative assessment; it should not be interpreted as the intrinsic density of the PLA material. A porosity fraction was not calculated because the filament material density and the actual printed material fraction were not recorded; the envelope-based apparent density therefore provides the available geometric normalization without introducing an assumed material density. Flexural-strength-to-mass ratio was also retained as a supplementary descriptive metric. Taken together with the flexural-strength results, these findings indicate that the core-related parameters accounted for most of the modelled variation in both responses. However, the factor levels associated with maximum flexural strength did not coincide with those associated with minimum mass. This response conflict provides the basis for the multi-response parameter selection procedure described in the following section.

### Contribution-weighted multi-response parameter selection

The response-specific ANOM results showed that maximum flexural strength and minimum mass favoured different levels of HCT, TFS, and BFS. A contribution-weighted procedure was therefore applied to combine the response-specific factor levels with the relative parameter contributions obtained from ANOVA. For each geometric parameter, the ANOVA contributions associated with flexural strength and mass were normalized by their sum. The resulting normalized values were used as response-specific weights for combining the factor levels identified by ANOM. In this manner, the response to which a parameter contributed more strongly exerted a correspondingly greater influence on the selected parameter value.

Table 7 presents the response-specific ANOVA contributions used in the procedure. The ANOM-selected factor levels and the response-specific contribution information were considered jointly in the matrix-based parameter-selection procedure within the investigated design space. The validation configuration retained in the experimental record and implemented in the CAD model had nominal dimensions of HCS = 6 mm, HCT = 1.08 mm, TFS = 1.18 mm, and BFS = 1 mm.

**Table 7:** Response-specific ANOVA contributions used in the multi-response parameter selection procedure.

			Honeycomb Cell Size		Honeycomb Cell Thickness		Top Face Sheet		Bottom Face Sheet	
			HCS		HCT		TFS		BFS	
**Responses**	**Flexural Strength (MPa)**	**FS**	${\text{P}}_{{\text{HCS}}_{FS}}$	45.92	${\text{P}}_{{\text{HCT}}_{FS}}$	34.07	${\text{P}}_{{\text{TFS}}_{FS}}$	8.88	${\text{P}}_{{\text{BFS}}_{FS}}$	0.06
	**Mass of the sandwich panel (g)**	**MA**	${\text{P}}_{{\text{HCS}}_{\text{MA}}}$	33.98	${\text{P}}_{{\text{HCT}}_{\text{MA}}}$	50.85	${\text{P}}_{{\text{TFS}}_{\text{MA}}}$	6.87	${\text{P}}_{{\text{BFS}}_{\text{MA}}}$	6.87
		**Total**	${\text{P}}_{{\text{HCS}}_{\text{T}}}$	79.90	${\text{P}}_{{\text{HCS}}_{\text{T}}}$	84.92	${\text{P}}_{{\text{TFS}}_{\text{T}}}$	15.75	${\text{P}}_{\text{B}{\text{FS}}_{\text{T}}}$	6.93

The response-specific ANOVA contributions were used to indicate the relative emphasis assigned to flexural strength and panel mass for each geometric parameter. For each geometric parameter, the contributions were normalized by their sum to obtain the response-specific weights:


$$\begin{array}{c} w_{j,FS}=\frac{P_{j,FS}}{P_{j,FS}+P_{j,MA}} \end{array}$$ (1)

$$\begin{array}{c} w_{j,MA}=\frac{P_{j,MA}}{P_{j,FS}+P_{j,MA}} \end{array}$$ (2)

Where $P_{j,FS}$ and $P_{j,MA}\;$denote the ANOVA percentage contributions of parameter to flexural strength and panel mass, respectively. Thus, $w_{j,FS}+\;w_{j,MA}=\text{1}\;$for each parameter. The normalized weights were used to represent the relative emphasis assigned to the two response objectives within the matrix-based selection procedure.

The normalized contribution ratios were considered together with the response-specific factor levels identified by ANOM to select a compromise configuration within the investigated design space. The procedure was used as a study-specific compromise-selection approach rather than as a predictive regression model or a global optimization algorithm. The complete intermediate numerical matrix calculation from the original parameter-selection procedure was not available in the archived experimental records accessible for this revision. Therefore, the intermediate numerical transformation leading to the final CAD dimensions is not reproduced here and has not been reconstructed using additional assumptions.

The final validation configuration retained in the experimental record and implemented in the validation CAD model had nominal dimensions of HCS = 6 mm, HCT = 1.08 mm, TFS = 1.18 mm, and BFS = 1 mm. The HCT and TFS values represent the nominal dimensions specified in the validation CAD model rather than post-print measured dimensions. Post-print dimensional measurements of HCT and TFS were not recorded. Because the extrusion-width and perimeter-count settings were also not retained, the precise sliced toolpaths used to realize these nominal intermediate dimensions cannot be reconstructed retrospectively.

### Experimental validation and performance comparison

**Fig 4:**
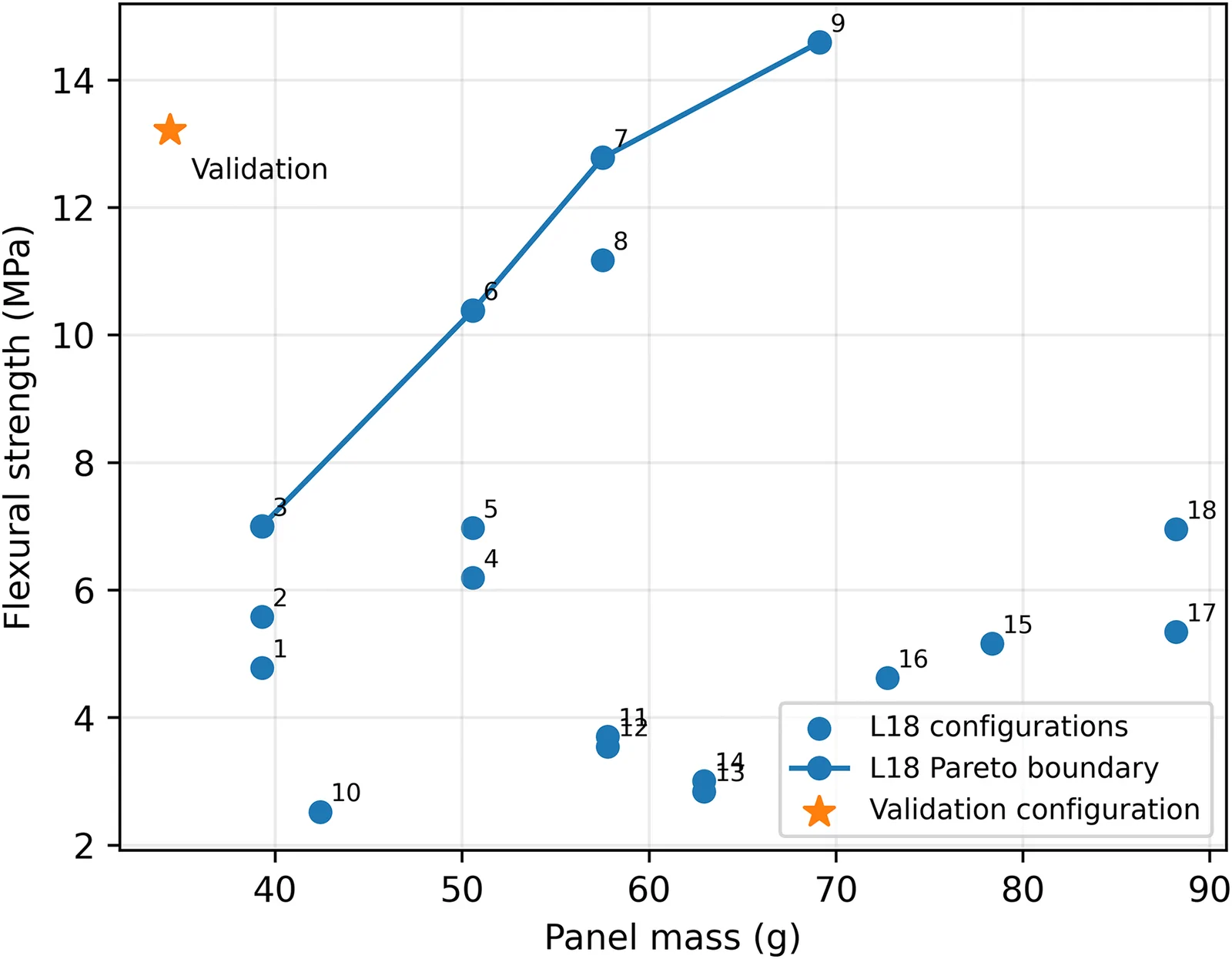
Strength–mass distribution of L18 and validation configurations with the L18 Pareto boundary.

The validation CAD configuration, with nominal dimensions of HCS = 6 mm, HCT = 1.08 mm, TFS = 1.18 mm, and BFS = 1 mm, was fabricated using the recorded printing conditions and evaluated under the same experimental procedure used for the L18 configurations. The validation experiment produced a flexural strength of 13.21 MPa and a panel mass of 34.367 g. These measured responses are associated with the validation CAD configuration recorded for the experiment; post-print measurements of the intermediate HCT and TFS dimensions were not available. Experiment 9 exhibited the highest flexural strength within the original L18 array, with 14.59 MPa at a mass of 69.13 g. Relative to Experiment 9, the validation configuration retained 90.54% of the measured flexural strength while reducing panel mass by 50.29%. Its measured mass was also lower than the minimum value of 39.29 g recorded among the original L18 configurations. These results indicate that the selected configuration provides a practical strength–mass compromise within the investigated design space rather than simply reproducing the optimum parameter combination for either individual response. For an additional descriptive comparison, the flexural-strength-to-mass ratio was calculated for the validation configuration and selected L18 configurations. Experiment 7 provided a ratio of 0.222 MPa/g, whereas Experiment 9, which had the highest measured flexural strength, provided 0.211 MPa/g. The validation configuration provided a ratio of 0.384 MPa/g. This comparison indicates that the selected configuration achieved a higher flexural-strength-to-mass ratio than these representative L18 configurations. The ratio is used here as a supplementary descriptor rather than as an additional optimization objective. This comparison is descriptive and does not establish global Pareto optimality beyond the investigated L18 configurations and the single validation configuration. For a further assessment of the strength–mass trade-off, the L18 results were examined using a Pareto-dominance comparison, with flexural strength treated as a quantity to be maximized and panel mass as a quantity to be minimized. The resulting strength–mass distribution is shown in Fig 4. Within the original L18 array, Experiments 3, 6, 7, and 9 formed the nondominated set. The validation configuration was also nondominated relative to the investigated L18 configurations because it provided higher flexural strength than the lower-strength, lower-mass configurations while maintaining a lower mass than the higher-strength configurations. This comparison is limited to the investigated design space and is presented to assess the practical position of the selected configuration rather than to establish global optimality. A formal prediction interval was not calculated because the available experimental record does not contain replicate observations or a separately fitted predictive model for the validation configuration. The validation result is therefore treated as an experimental check of the selected configuration rather than as a statistical prediction of its response.

## Conclusions

This study experimentally investigated the effects of honeycomb cell size, cell-wall thickness, top face-sheet thickness, and bottom face-sheet thickness on the flexural strength and mass of FFF-printed PLA honeycomb sandwich panels. A mixed-level Taguchi L18 design, ANOM, ANOVA, and a contribution-weighted multi-response procedure were used to evaluate the geometric effects and select a compromise configuration. The principal findings are as follows:

- Within the investigated design space, the core-related variables accounted for most of the modelled variation in flexural strength. HCS and HCT contributed 45.92% and 34.07%, respectively, although the HCS contribution includes the associated change in overall specimen geometry.
- HCT was the dominant parameter affecting panel mass, with a contribution of 50.85%, followed by HCS at 33.98%. The results demonstrate that the parameters most strongly affecting mechanical performance also substantially influence material usage.
- TFS contributed 8.88% to the variation in flexural strength, although its effect was borderline at the 5% significance level (p = 0.053), whereas BFS showed no statistically significant effect on flexural strength within the investigated parameter range.
- The study-specific multi-response selection procedure was used to select a validation CAD configuration with nominal dimensions of HCS = 6 mm, HCT = 1.08 mm, TFS = 1.18 mm, and BFS = 1 mm. The experimentally evaluated validation configuration yielded a flexural strength of 13.21 MPa and a mass of 34.367 g. Relative to Experiment 9, which exhibited the highest measured flexural strength in the L18 array, the validation result retained 90.54% of the flexural strength while reducing panel mass by 50.29%.
- The validation experiment indicates that the study-specific multi-response parameter-selection procedure can provide a practical strength–mass compromise within the investigated design space. Because only one validation specimen was tested, the robustness and repeatability of the selected configuration require further experimental verification.

The findings are limited to the PLA material, fabrication conditions, geometric ranges, and three-point bending arrangement investigated. Because the HCS levels were accompanied by changes in overall specimen length and support span, the present design does not isolate the effect of cell size from the associated specimen geometry. In addition, the main-effects analysis does not quantify parameter interactions. One specimen was tested for each L18 configuration and one specimen for the confirmation configuration; therefore, experimental repeatability could not be quantified through replicate testing. Future studies should therefore use geometrically normalized specimens, replicate testing, broader printing conditions, and experimental designs capable of resolving relevant parameter interactions.

## Supporting information

S1 File Geometric design, specimen dimensions, Taguchi L18 experimental design, measured responses, and validation result used in the present study. (DOCX)
