# Supplementary material for: Experimental investigation and multi-response design of FFF-printed PLA honeycomb sandwich panels for flexural performance and mass efficiency
Source: PLoS One. 2026 Sep 21;21(9):e0358969. doi: 10.1371/journal.pone.0358969 (PMC13592624; doi:10.1371/journal.pone.0358969)
Supplement: Supplementary file 1 [file pone.0358969.s001.docx]

**S1. CAD Modelling and Specimen Preparation**

All honeycomb sandwich panels were modelled using Autodesk Fusion 360 (2024). The models were exported in STL format and processed using Simplify3D software to generate G-code for fabrication.

A conventional hexagonal honeycomb topology was maintained throughout the study. Only the following geometric parameters were varied according to the Taguchi L18 orthogonal array:

- Honeycomb cell size (HCS)
- Honeycomb cell-wall thickness (HCT)
- Top face-sheet thickness (TFS)
- Bottom face-sheet thickness (BFS)

All other printing conditions were maintained constant. The HCT and TFS values of the validation configuration represent the nominal dimensions specified in the validation CAD model.

**S2. Printing Parameters**

Printing material: Commercial PLA filament (1.75 mm nominal diameter), manufactured by eSUN; exact grade/type was not recorded.
Printer: PRATHAM 5.0 FFF Printer (MAKE3D.IN)
Nozzle diameter: 0.4 mm
Extrusion temperature: 210 °C
Layer thickness: 0.20 mm
Infill density: 100%
Printing speed: 30 mm/s
Bed temperature: 55 °C
Cooling fan: Off
Wall/perimeter count: Not recorded
Extrusion width: Not recorded
Filament conditioning: The filament was used as received from the supplier, without additional drying, reheating, or pre-conditioning.
Printing orientation: Flat orientation, with the bottom face placed directly on the build plate. The longitudinal direction was aligned with the X-axis of the build platform and the honeycomb cell walls were built vertically along the Z-axis.

**S3. Three-Point Bending Test**

Three-point bending tests were carried out using a Universal Testing Machine equipped with a 10 kN load cell.

Testing conditions:

- Loading mode: Three-point bending
- Crosshead speed: 2 mm/min
- Loading nose diameter: 30 mm
- Span length: Adjusted according to specimen height to maintain an approximate span-to-height ratio of 3, as reported in Table 2 of the manuscript.

The maximum load obtained during the three-point bending test was used to determine the reported flexural strength. The exact flexural-strength calculation expression and individual maximum-load values were not available among the experimental records accessible for this revision. Consequently, no additional calculation assumptions were introduced. One specimen was tested for each L18 configuration, and one specimen was tested for the validation configuration.

**S4. Experimental Responses**

The following responses were measured for each specimen:

- Flexural strength (MPa)
- Panel mass (g)

Flexural strength was treated as a larger-the-better response.

Mass was treated as a smaller-the-better response.

**S5. Statistical Analysis**

The experimental data were analysed using:

- Taguchi L18 orthogonal array
- Analysis of Means (ANOM)
- Analysis of Variance (ANOVA)

ANOM was used to determine the preferred factor levels for each response independently.

ANOVA was used to determine the percentage contribution and statistical significance of each geometric parameter.

**S6. Multi-Response Parameter Selection**

The response-specific ANOM results and ANOVA contribution percentages were used in the matrix-based multi-response parameter-selection procedure adopted in the original experimental programme. The procedure was used as a study-specific compromise-selection approach within the investigated factor ranges. The complete intermediate numerical matrix output from the original calculation was not available in the archived experimental documentation used for the present revision and was therefore not reconstructed using additional assumptions.

The final validation CAD configuration retained in the experimental record had the following nominal dimensions:

• HCS = 6 mm

• HCT = 1.08 mm

• TFS = 1.18 mm

• BFS = 1 mm

The HCT and TFS values represent nominal dimensions specified in the validation CAD model rather than post-print measured dimensions.

**S7. Validation Experiment**

The selected validation CAD configuration was fabricated using the recorded printing conditions and evaluated under the same experimental procedure used for the L18 configurations. The nominal validation CAD dimensions were HCS = 6 mm, HCT = 1.08 mm, TFS = 1.18 mm, and BFS = 1 mm.

Post-print dimensional measurements of HCT and TFS were not recorded in the available experimental documentation. Because the extrusion-width and wall/perimeter-count settings were also not retained, the precise sliced toolpaths used to realize the nominal 1.08 mm HCT and 1.18 mm TFS dimensions cannot be reconstructed retrospectively.

Measured responses:

• Flexural strength = 13.21 MPa

• Panel mass = 34.367 g
